# Unsupervised Detection of Suspicious Tissue Using Data Modeling and PCA

**DOI:** 10.1155/IJBI/2006/57850

**Published:** 2006-07-11

**Authors:** Ikhlas Abdel-Qader, Lixin Shen, Christina Jacobs, Fadi Abu Amara, Sarah Pashaie-Rad

**Affiliations:** ^1^ Department of Electrical and Computer Engineering, Western Michigan University, MI 49008, USA; ^2^ Department of Mathematics, Western Michigan University, Kalamazoo, MI 49008, USA; ^3^ Radiology Department, Bronson Methodist Hospital, Kalamazoo, MI 49007, USA; ^4^ Automated Test Engineering Department, Saint Jude Medical, Valencia, CA 91381, USA

## Abstract

Breast cancer is a major cause of death and morbidity among women all over the world, and it is a fact that early detection is a key in improving outcomes. Therefore development of algorithms that aids radiologists in identifying changes in breast tissue early on is essential. In this work an algorithm that investigates the use of principal components analysis (PCA) is developed to identify suspicious regions on mammograms. The algorithm employs linear structure and curvelinear modeling prior to PCA implementations. Evaluation of the algorithm is based on the percentage of correct classification, false positive (FP) and false negative (FN) in all experimental work using real data. Over 90% accuracy in block classification is achieved using mammograms from MIAS database.


Hindawi Publishing Corporation
International Journal of Biomedical Imaging
Volume 2006, Article ID 57850, Pages 1–11
DOI 10.1155/IJBI/2006/57850

### Unsupervised Detection of Suspicious Tissue Using Data Modeling and PCA

**Ikhlas Abdel-Qader,[1] Lixin Shen,[2] Christina Jacobs,[3] Fadi Abu Amara,[1] and Sarah Pashaie-Rad[4]**

[1] *Department of Electrical and Computer Engineering, Western Michigan University, MI 49008, USA*
[2] *Department of Mathematics, Western Michigan University, Kalamazoo, MI 49008, USA*
[3] *Radiology Department, Bronson Methodist Hospital, Kalamazoo, MI 49007, USA*
[4] *Automated Test Engineering Department, Saint Jude Medical, Valencia, CA 91381, USA*

Received 22 January 2006; Revised 20 May 2006; Accepted 6 June 2006

Breast cancer is a major cause of death and morbidity among women all over the world, and it is a fact that early detection is a key in improving outcomes. Therefore development of algorithms that aids radiologists in identifying changes in breast tissue early on is essential. In this work an algorithm that investigates the use of principal components analysis (PCA) is developed to identify suspicious regions on mammograms. The algorithm employs linear structure and curvelinear modeling prior to PCA implementations. Evaluation of the algorithm is based on the percentage of correct classification, false positive (FP) and false negative (FN) in all experimental work using real data. Over 90% accuracy in block classification is achieved using mammograms from MIAS database.

Copyright © 2006 Ikhlas Abdel-Qader et al. This is an open access article distributed under the Creative Commons Attribution License, which permits unrestricted use, distribution, and reproduction in any medium, provided the original work is properly cited.

### 1. INTRODUCTION

Breast cancer is the second leading cause of cancer death in women in the United States. Early detection is crucial for treatment success as tumor size is a major prognostic indicator. Studies have shown that early detection and treatment improve the chances of survival for breast cancer patients [1, 2]. The American Cancer Society recommends all women 40 years and older undergo yearly screening mammograms. The goal of screening mammography is the detection of cancer before it becomes palpable. Unfortunately, mammograms are not 100% accurate. False positive rates of 15–30% and false negative rates of 10–30% have been reported [3]. False-positives (labeling a finding as suspicious which later is found to be benign) lead to unnecessary biopsies and anxiety, while false-negatives (failure to detect presence of cancer) result in later detection and often poorer prognosis. Nonetheless, mammography has an overall accuracy rate of 90% [2].

Although radiologists are capable of detecting a number of findings suggesting cancerous tissues in radiographic images, a significant percentage of abnormalities are missed [3]. Screening programs typically require radiologists to read large numbers of mammograms with great attention to fine details. As less than 10% of exams will have abnormalities that need further attention and only around 1% will actually have cancer, this is a rather tedious process. Fatigue, satisfaction of search (failing to detect additional abnormalities once one finding is detected), and failure to perceive subtle changes are common causes of false negative exams. Development of computer algorithms to assist radiologists in detection of abnormalities would be extremely beneficial. Masses can be hidden by normal dense glandular tissue and fine microcalcifications can blend in with background tissue. Computer-aided image analysis enables detection of mass-like structures only a few millimeters in size and even smaller microcalcifications. Cancerous tissues usually arise in duct channels and lobules. It is critical to define the degree of abnormality compared to normal cells and growth rate of abnormal cells, which is named tumor grade.

Computerized feature extraction techniques are used to extract features in mammographic images that may not be readily perceived by radiologists. Many methods have been proposed in the literature for mammography detection and classification utilizing a wide variety of algorithms to achieve their goals. Chan et al. [4] used artificial neural networks to extract features from mammograms to predict whether the presence of microcalcifications is associated with malignant or benign pathology. A back-propagation artificial neural network classifier was trained and tested with a leave-one-case-out method to recognize the malignant or benign microcalcification clusters. 11 out of the 28 benign cases were correctly identified (39%) without missing any malignant cases. Lemaur et al. [5] used wavelets having a high Sobolev regularity to detect clustered microcalcification in digitized mammograms. Morrow et al. [6] used each pixel in the mammographic images as a seed to grow a region. Then, the contrast of each region is calculated and enhanced by applying an empirical transformation based on each region's seed pixel value, its contrast, and its background. The validity of microcalcification clusters and anatomic details is considerably improved in the processed images. Shen et al. [7] developed a set of shape factors to measure the roughness of contours of calcifications in mammograms and for use in microcalcification classification as malignant or benign. Wang and Karayiannis [8] used wavelet transform to decompose the mammograms into different frequency subbands. They suppressed the low-frequency subbands, making microcalcifications correspond to high-frequency components, and reconstructed the mammogram from the subbands containing only high frequencies. Zwiggelaar [3] used fractals and statistical modeling to separate the structure and texture background that are present in mammographic images.

Utilizing PCA and feature modeling in this work is to target identifying abnormal mammograms in a local processing setting. It identifies the region of suspicious tissues in an area size of $120 \times 120$. This paper is organized as follows. Section 2 presents a procedure of PCA. Section 3 presents the linear and curvelinear modeling of the data. Simulation results are presented in Section 4 followed by the conclusions in Section 5.

### 2. BACKGROUND ON PCA

Principal component analysis has proven to be one of the best methods to find similar patterns or features in a data set. It is an essential statistical tool in pattern recognition applications which includes medicine (such as sample identification), industry (quality control and document image analysis), and government (fingerprint identification and face recognition) [9–11]. Ye and Auner [11] used PCA to reduce the dimensionality of signatures for different types of samples to create a real-time approach of sample analysis using a Raman spectrometer directly mounted at the end-effector of medical robot to enhance the remote robot surgery. Chen et al. [9] developed algorithms based on PCA to generate a set of new identifying keys for a given set of patterns to reduce the number of comparisons during the near-matching process. Pinkowski [10] used PCA for feature reduction on a speaker-dependent data set to achieve high recognition rate analyzing spectrograms, which contain human speech utterances. 97.5% correct recognition rate is achieved using PCA. Also, Swiniarski and Swiniarsk [12] used PCA with rough set methods for feature selection in mammograms and reported good results.

In general, PCA algorithm can be useful whenever an automated feature extraction or identification from a digital image is required. It is based on finding a match for the specific feature in the test image from image database using some similarity measures. These measures are defined based on statistical characteristics of the data: variance, covariance, eigenvectors, and eigenvalues. A brief description of PCA is given in the following subsection and followed by an introduction of two distance measures used in this work.

### 2.1. A brief description of PCA

Suppose that we have $m$ training data vectors $\mathbf{x}_1, \mathbf{x}_2, \ldots, \mathbf{x}_m$ of $n$ dimensions each, that is, $\mathbf{x}_j = [x_{1j}, \ldots, x_{nj}]^T$. There are two phases in the algorithm of PCA. The first phase is to find $p$ orthogonal and uncorrelated vectors and the second phase is to project the given data set into a subspace spanned by these $p$ orthogonal vectors.

The first phase of PCA is as follows.

(i) Construct an $n \times 1$ vector $\mathbf{m}$ whose $i$th element $m_i$ is the mean of the $i$th dimension of all data $\mathbf{x}$, that is,

$$m_i = \frac{1}{m} \sum_{k=1}^{m} x_{ik}. \tag{1}$$

(ii) Form an $n \times m$ matrix $\mathbf{X} = [\mathbf{x}_1 - \mathbf{m}, \mathbf{x}_2 - \mathbf{m}, \ldots, \mathbf{x}_m - \mathbf{m}]$, that is, $[\mathbf{X}]_{ij} = x_{ij} - m_i$.

(iii) The $n \times n$ covariance matrix $\mathbf{C}$ of $\mathbf{X}$ is

$$\mathbf{C} = \mathbf{X}\mathbf{X}^T. \tag{2}$$

(iv) Let $\lambda_i$ and $\mathbf{v}_i$, $i = 1, 2, \ldots, n$, be eigenvalues and normalized eigenvectors of the matrix $\mathbf{C}$ with $\mathbf{C}\mathbf{v}_i = \lambda_i \mathbf{v}_i$ and $\lambda_1 \geq \lambda_2 \geq \cdots \geq \lambda_n \geq 0$. Eigenvectors are called the principal components.

Note that the covariance matrix $\mathbf{C}$ is symmetric and semidefinite. We have $\mathbf{v}_i^T \cdot \mathbf{v}_j = 0$ for all $\lambda_i \neq \lambda_j$. If $\lambda_i$ is the repeated eigenvalue of $\mathbf{C}$, the associated principle component $\mathbf{v}_i$ is not unique.

The second phase of PCA is to project a given $n \times 1$ testing data $\mathbf{x}$ into a space spanned by $\mathbf{v}_1, \mathbf{v}_2, \ldots, \mathbf{v}_p$, the eigenvectors associated with the first $p$ largest eigenvalues of the covariance matrix $\mathbf{C}$. This space is called eigenspace. The projection of the testing data $\mathbf{x}$ on the eigenspace is

$$\mathbf{y} = \mathbf{P}^T(\mathbf{x} - \mathbf{m}), \tag{3}$$

where $\mathbf{P} = [\mathbf{v}_1 \ \ \mathbf{v}_2 \ \ \cdots \ \ \mathbf{v}_p]$.

Discarding small eigenvalues, and consequently corresponding eigenvectors, results in dimension reduction and increases the speed of computations. The highest eigenvalues are associated with the eigenvectors that contain the major modes of variation in the data. Once test mammogram and training set all projected in the eigenspace, a distance measure is used to find the nearest match of the test mammogram to the mammogram in the training set. Approximately 90% of the total variance is contained in the first 5 to 10 of the dimensions. In most implementations of PCA for feature extraction applications, the important decision is how much of the original data variation is needed to be captured in the eigen features. Most researches choose to work with 88% to 98% of original data variations (see [11]). Including all of the principal components would be equivalent to working with the original data since each one of these vectors is a linear combination of the original vectors. It is worth mentioning that keeping the first few components and discarding the others will result in a loss of the original data, however, in this utilization of the PCA, this loss is insignificant due to the fact that the principal components are used as feature vectors to match them with the feature vectors from mammograms in the training set. The original mammogram is preserved and the output for the radiologist is the original mammogram with identified blocks as suspicious block.

Thresholding is implemented by throwing away all eigenvalues that are below a threshold $T$, which is basically a measure of how much variation in the original data is accounted for in the eigenvalues and their corresponding eigenvectors that are preserved. The threshold $T$ is calculated as follows:

$$T = \frac{\sum_{k=1}^{L} \lambda_k}{\sum_{k=1}^{n} \lambda_k}, \tag{4}$$

where $L$ is usually kept much smaller than the original total number of the eigenvalues $n$. In our simulation, we choose $L = 36$.

### 2.2. *Distance measurements*

A common way of finding similarities between two patterns is to find the difference. Since minimum distance means maximum similarity, different types of distance measurements are being used. In this study, two types are employed: Euclidean and Chebyshev with the intention to compare them and find the optimum one for mammographic data.

(i) *Euclidean distance*

This type of distance is the standard metric, which is the shortest distance between two vectors ($\mathbf{x}$ and $\mathbf{y}$) and is defined as follows:

$$d_e(\mathbf{x}, \mathbf{y}) := \sqrt{\sum_i \left(x_i - y_i\right)^2}. \tag{5}$$

(ii) *Chebyshev distance*

This type of distance is also known as maximum value distance. It examines the absolute magnitude of the differences between coordinates of a pair of objects. When computation time is extremely imperative, Chebyshev distance is used. It is defined as follows:

$$d_{\text{ch}}(\mathbf{x}, \mathbf{y}) := \max_i \left| x_i - y_i \right|. \tag{6}$$

### 3. MODELING OF IMAGE FEATURES

Initially the intention was to identify the image features using a linear structure model in an effort to improve the results obtained by utilizing PCA alone [13]. Indeed, a proper choice of a modeling technique that suites the features in the data can be combined with PCA to improve results as reported in [10, 11, 13]. In this work, the search is for suspicious tissue which can be identified by regions surrounded by an edge. Edges in images can be detected using several algorithms such as directional morphology, curve-linear structure detection, directional second-order Gaussian derivative, and convolution-based edge detection algorithms [14–21]. Also, edges may have several shapes such as straight lines, circles, ellipses, and parabola. Thus it is important that a suitable data modeling is utilized to better fit the nature of the features captured in the data.

### 3.1. *Linear modeling*

Convolution-based edge detector is used in this work. Four masks with the size of $5 \times 5$ pixels are used for vertical, horizontal, and oblique ($\pm 45^\circ$) structure detection such as

$$R(n_1, n_2) = \sum_{k_1=-2}^{2} \sum_{k_2=-2}^{2} a(k_1, k_2) b(n_1 - k_1, n_2 - k_2), \tag{7}$$

where $R$ is the resultant image, $a$ is the mask, and $b$ is the input image. When a vertical mask is convolved with an image, the longitudinal linear structures are extracted in the resultant image. Using other masks results in similar outputs. Once all edges in all directions are identified, an edge mammogram is generated. It is more common to use mask size of $3 \times 3$ and they are seldom to be greater than $7 \times 7$, we decide to use $5 \times 5$. Masks used in this implementation for detecting vertical, horizontal, oblique with $45^\circ$ and $-45^\circ$ edges are

$$\begin{bmatrix} -1 & -1 & 4 & -1 & -1 \\ -1 & -1 & 4 & -1 & -1 \\ -1 & -1 & 4 & -1 & -1 \\ -1 & -1 & 4 & -1 & -1 \\ -1 & -1 & 4 & -1 & -1 \end{bmatrix}, \quad \begin{bmatrix} -1 & -1 & -1 & -1 & -1 \\ -1 & -1 & -1 & -1 & -1 \\ 4 & 4 & 4 & 4 & 4 \\ -1 & -1 & -1 & -1 & -1 \\ -1 & -1 & -1 & -1 & -1 \end{bmatrix},$$

$$\begin{bmatrix} -1 & -1 & -1 & -1 & 4 \\ -1 & -1 & -1 & 4 & -1 \\ -1 & -1 & 4 & -1 & -1 \\ -1 & 4 & -1 & -1 & -1 \\ 4 & -1 & -1 & -1 & -1 \end{bmatrix}, \quad \begin{bmatrix} 4 & -1 & -1 & -1 & -1 \\ -1 & 4 & -1 & -1 & -1 \\ -1 & -1 & 4 & -1 & -1 \\ -1 & -1 & -1 & 4 & -1 \\ -1 & -1 & -1 & -1 & 4 \end{bmatrix}, \tag{8}$$

respectively.

### 3.2. *Curvilinear modeling*

Hough Transform (HT) was introduced by Hough in 1962 for detecting and recognizing complex patterns in data [22].

HT does not require connected or even nearby edge points. Literature is rich in articles on HT and its abilities in tracking edges, lines, curves, and parabolic features in images. Fundamentally it is a mapping process of edge pixels into a parameter space [22, 23]. Edges that represent straight lines, for example, are identified with their slope-intercept $(m, b)$ parameters and the line for edge pixel $(x, y)$ is

$$y = mx + b. \tag{9}$$

However, due to the difficulty dealing with vertical lines in the image space, a different description for straight line is commonly used [23]. Lines are parameterized by the orientation of the line $\theta$ and the distance of the line from the origin $\rho$ as follows:

$$\rho = (x_i - x_0)\cos\theta + (y_i - y_0)\sin\theta, \tag{10}$$

where all points $(x_i, y_i)$ of an edge in the image space are represented by $(\theta, \rho)$ point in the transform space, and $(x_0, y_0)$ is the origin of the image space. While this algorithm has proved its efficiency in applications where features are straight lines, however, suspicious regions tend to have circular shapes. Thus HT for curves is a mapping of a pixel point $(x_i, y_i)$ in an image space to a sinusoidal curvilinear in the Hough space $(\rho, \theta)$. A complete review of detecting Hough transforms curves can be found in [24, 25]. In general, curves of various sizes can be identified in the image scene by introducing a new parameter such as a radius of a circle $r$. The transform makes use of the circle formula

$$(x - x_0)^2 + (y - y_0)^2 = r^2 \tag{11}$$

to find the pixels that fall on this circle and simultaneously increases the particular accumulator positions.

HT can be high in computational cost and complexity and has some disadvantages such as the fact that some lines are replicated during detection due to spatial sampling. Nevertheless, HT is a powerful tool for detecting features with various sizes and orientations with high accuracy and research is ongoing in developing computationally efficient algorithms [26]. Recently, Olson in [27] has presented superior algorithm to provide accurate and fast Hough curves with a worst-case complexity of $O(n)$.

### 4. EXPERIMENTAL RESULTS

The algorithm outlined above was simulated on mammograms from the Mammographic Image Analysis Society (MIAS) database using Matlab . The simulations can be categorized as follows: the first is utilizing PCA with linear modeling in local processing, and the second is utilizing PCA with HT as a method for curvilinear modeling. In a global approach one would consider each mammogram is an image as where in local processing each mammogram is segmented into blocks prior to any processing and each block is processed as it was an image. Instead of a training set of mammograms, we are training a set of blocks from several mammograms. Earlier work showed that PCA using a local processing is performing better [13]. This is an attempt to examine PCA performance in a small neighborhoods that may contain some mamographic features of interest.

Table 1: Decision table for training set segments.

| Training set | | Segments | | | | | | | | | | | | Image is |
|---|---|---|---|---|---|---|---|---|---|---|---|---|---|---|
| | | 1 | 2 | 3 | 4 | 5 | 6 | 7 | 8 | 9 | 10 | 11 | 12 | |
| 1 | mdb001 | n | n | n | n | s | n | s | s | s | n | n | n | **S** |
| 2 | mdb002 | n | n | n | n | n | n | s | s | s | n | s | s | **S** |
| 3 | mdb003 | n | n | n | n | n | n | n | n | n | n | n | n | **N** |
| 4 | mdb004 | n | n | n | n | n | n | n | n | n | n | n | n | **N** |
| 5 | mdb058 | n | n | n | n | n | n | s | n | n | n | n | n | **S** |
| 6 | mdb072 | n | n | n | n | n | n | n | s | n | n | s | n | **S** |
| 7 | mdb008 | n | n | n | n | n | n | n | n | n | n | n | n | **N** |
| 8 | mdb009 | n | n | n | n | n | n | n | n | n | n | n | n | **N** |
| 9 | mdb012 | n | n | n | n | s | n | n | n | n | n | n | n | **S** |
| 10 | mdb013 | n | n | n | n | n | n | n | s | s | n | n | n | **S** |
| 11 | mdb075 | n | n | n | n | s | n | n | n | n | n | n | n | **S** |
| 12 | mdb090 | n | n | n | s | s | n | s | s | n | n | n | n | **S** |

Twelve images are segmented into 12 blocks each resulting in 144 elements trained. These mammograms are combinations of normal, benign, and malignant mammograms. All these images/blocks from MIAS database are labeled and their information are tabulated in Table 1. Table 1 is the decision table, where the status of each block is visually inspected and labeled by a radiologist. As for the blocks, each block is defined to be either normal (n) or suspicious (s). Figure 1 shows one of the images of each group. Figure 1(a) is a benign mammogram in training set database, the area around nipple is defined to be suspicious. Tissues of interest are referred to as suspicious in this paper, whether they are malignant or benign. Making final decision is left to a specialist. Figure 1(b) is a normal mammogram in the training set database. Figure 1(c) is a malignant mammogram, and areas around milk ducts are suspicious. The local processing can be computationally expensive depending on the training set size and number of blocks in each image. Moreover, adding more mammograms to the training set increases memory requirements.

Fifteen mammograms were used as testing set, which mounts to 180 block testing and matchings results. Figure 2 shows three samples of test images. Each of them belongs to one of the three classified groups: benign, normal, and malignant, respectively.

### 4.1. *Results from linear structure modeling*

This algorithm is based on extracting the linear models in each image or block using a convolution process. The detectors are composed of four 5 × 5 masks responding to vertical, horizontal, and oblique (±45°) lines. This method

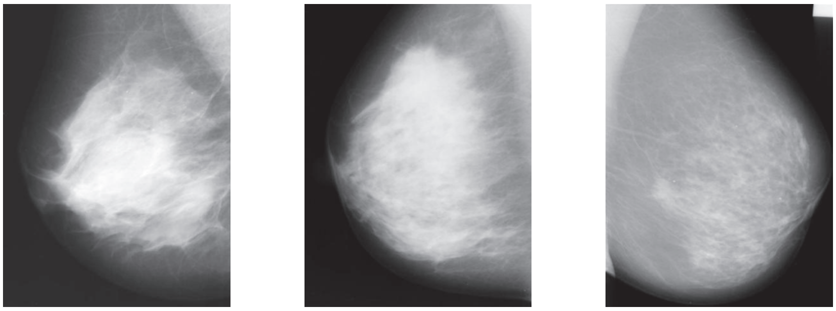

Figure 1: (a) Benign mammogram in training set mdb001; (b) normal mammogram in training set mdb003; and (c) malignant mammogram in training set mdb058.

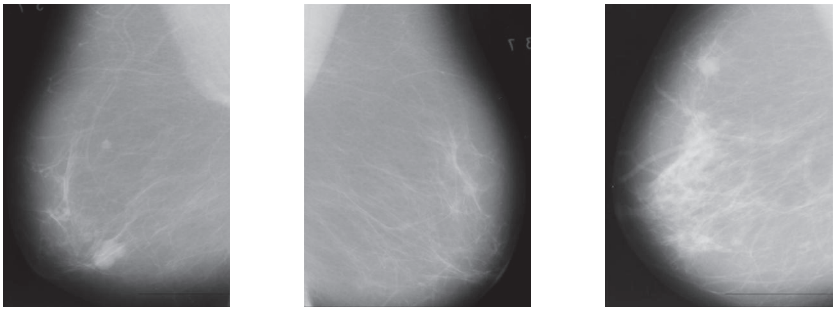

Figure 2: (a) Benign mammogram in testing set mdb005; (b) normal mammogram in testing set mdb006; and (c) malignant mammogram in testing set mdb023.

was applied to 15 randomly selected images such as the ones displayed in Figure 2 using both Euclidean distance and Chebyshev distance. Total number of testing blocks are 180 and total number of trained blocks are 144.

Results with Euclidean and Chebyshev distance measures are tabulated in Tables 2 and 3, respectively. These results indicate that Euclidian distance is capable of achieving similar results to Chebyshev distance in terms of a mammogram classification as suspicious or not, both at 60% correct, 26.6% FP, and 13.3% FN. Out of the fifteen tests performed, 9 were correct, 4 were FP, and 2 FN using both distance measures. However, the Euclidian distance has shown much higher accuracy with PCA using block statistics. That is, in the Euclidean distance simulations, 159 (88.33%) blocks were correctly classified, while 9 (5%) FP, and 12 (6.66%) FN classifications. As for the Chebyshev results they are at 157 (87.22%) blocks correctly classified, 11 (6.11%) FP, and 12 (6.66%) FN classifications. The Euclidean distance has a comparable accuracy to Chebyshev's with the first has two less FP block classification which requires less viewing time by the radiologist.

### 4.2. Results from curvilinear modeling

The results of using HT transform as an algorithm to identify the curvilinear features in the mammogram as opposed to straight edges are displayed in Tables 4 and 5.

From these tables, it is observed that the correct mammogram classifications is improved to 73.3% with 20% FP and 6.67% FN classifications for the Eculidean disteance while the results of the Chebyshev distance are improved to 66.67% correct classifications, and 26.67% FP, and 6.67% FN. However, since the objective here is to alert a radiologist to suspicious regions in the test mammogram, it is more proper again to look at the block classification results. These are at 91.11% correct classifications, 3.33% FP, and 5.55% FN for the Euclidean distance measure while the Chebyshev results are at 90% correct block classifications, 4.44% FP, and 5.55% FN. It is worth noting that mdb016 was consistently classified as suspicious mammogram when it is a normal mammogram. This is due to the fact that this image is a mammogram of fatty and glandular tissue. Tissue composition ion mammography is important as the detection of cancer is easier in fatty tissue and mammography becomes less sensitive the more dense tissue (the greater the proportion of fibroglandular tissue). While this mammogram is not suspicious, it is not uncommon for a CAD system to mark an area of normal overlapping tissue as suspicious since its tissue is highly glandular tissue.

### 5. CONCLUSION

Our goal is to detect abnormalities in screening mammograms as an additional support system to assist radiologists.

Table 2: Results using block processing with Euclidean distance. Note: first number indicates the block number and second one signifies the image number in training set.

| Testing set | | Segments | | | | | | | | | | | | Image is |
|---|---|---|---|---|---|---|---|---|---|---|---|---|---|---|
| | | 1 | 2 | 3 | 4 | 5 | 6 | 7 | 8 | 9 | 10 | 11 | 12 | |
| mdb005 | **S** | 11,10 | 5,10 | 9,10 | 11,11 | 5,11 | 9,8 | 2,3 | 8,7 | 9,8 | 11,11 | 11,8 | 12,3 | **S** |
| | | n | n | s | n | s | n | n | n | n | n | n | n | |
| mdb006 | **N** | 1,7 | 8,5 | 9,7 | 4,4 | 8,9 | 9,5 | 7,7 | 8,7 | 9,7 | 10,4 | 2,2 | 11,6 | **S** |
| | | n | n | n | n | n | n | n | n | n | n | n | s | |
| mdb007 | **N** | 1,10 | 2,3 | 6,8 | 12,12 | 5,8 | 9,8 | 7,11 | 8,8 | 9,8 | 10,3 | 11,3 | 12,3 | **N** |
| | | n | n | n | n | n | n | n | n | n | n | n | n | |
| mdb010 | **S** | 1,12 | 2,12 | 6,4 | 7,6 | 5,5 | 6,4 | 7,6 | 8,5 | 9,5 | 10,6 | 12,10 | 12,12 | **N** |
| | | n | n | n | n | n | n | n | n | n | n | n | n | |
| mdb023 | **S** | 10,3 | 2,1 | 3,8 | 10,3 | 5,10 | 6,10 | 7,1 | 2,11 | 6,10 | 10,3 | 8,1 | 9,8 | **S** |
| | | n | n | n | n | n | n | s | n | n | n | s | n | |
| mdb028 | **S** | 1,7 | 2,12 | 6,7 | 4,2 | 5,9 | 9,5 | 4,2 | 8,9 | 12,7 | 10,5 | 11,9 | 9,5 | **S** |
| | | n | n | n | n | s | n | n | n | n | n | n | n | |
| mdb011 | **N** | 0,3 | 2,1 | 3,8 | 7,1 | 5,10 | 6,10 | 10,10 | 8,11 | 12,8 | 10,3 | 7,1 | 3,7 | **S** |
| | | n | n | n | s | n | n | n | n | n | n | s | n | |
| mdb014 | **N** | 1,7 | 5,6 | 3,7 | 1,5 | 8,12 | 6,7 | 7,4 | 8,5 | 9,5 | 10,7 | 11,12 | 12,12 | **S** |
| | | n | n | n | n | s | n | n | n | n | n | n | n | |
| mdb016 | **N** | 4,12 | 5,5 | 12,1 | 7,12 | 8,9 | 9,5 | 4,12 | 8,5 | 12,7 | 7,1 | 11,6 | 12,12 | **S** |
| | | s | n | n | s | n | n | s | n | n | s | s | n | |
| mdb015 | **S** | 10,11 | 5,10 | 6,11 | 7,1 | 5,11 | 3,1 | 7,1 | 8,11 | 6,1 | 4,11 | 6,4 | 3,12 | **S** |
| | | n | n | n | s | s | n | s | n | n | n | n | n | |
| mdb017 | **S** | 12,12 | 5,3 | 2,6 | 4,3 | 5,3 | 9,1 | 12,12 | 8,1 | 9,7 | 4,1 | 11,10 | 12,7 | **S** |
| | | n | n | n | n | n | s | n | s | n | n | n | n | |
| mdb019 | **S** | 1,10 | 2,1 | 3,8 | 11,11 | 5,11 | 3,10 | 2,10 | 8,7 | 6,10 | 2,3 | 8,7 | 12,8 | **S** |
| | | n | n | n | n | s | n | n | n | n | n | n | n | |
| mdb095 | **S** | 7,1 | 2,1 | 9,10 | 12,12 | 5,1 | 6,8 | 7,1 | 5,11 | 9,8 | 7,11 | 11,8 | 12,10 | **S** |
| | | s | n | s | n | s | n | s | s | n | n | n | n | |
| mdb102 | **S** | 1,12 | 2,12 | 12,11 | 7,6 | 5,6 | 6,4 | 1,12 | 2,12 | 3,12 | 10,6 | 12,10 | 3,12 | **N** |
| | | n | n | n | n | n | n | n | n | n | n | n | n | |
| mdb092 | **S** | 4,12 | 5,9 | 6,7 | 4,12 | 5,12 | 6,4 | 7,12 | 8,6 | 6,4 | 10,6 | 12,10 | 6,4 | **S** |
| | | s | s | n | s | s | n | s | s | n | n | n | n | |

Table 3: Results using block processing with Chebyshev distance. Note: first number indicates the block number and second one signifies the image number in training set.

| Testing set | | Segments | | | | | | | | | | | | Image is |
|---|---|---|---|---|---|---|---|---|---|---|---|---|---|---|
| | | 1 | 2 | 3 | 4 | 5 | 6 | 7 | 8 | 9 | 10 | 11 | 12 | |
| mdb005 | **S** | 11,10 | 5,10 | 9,10 | 11,11 | 5,11 | 9,8 | 1,8 | 8,7 | 9,8 | 11,11 | 2,4 | 12,3 | **S** |
| | | n | n | s | n | s | n | n | n | n | n | n | n | |
| mdb006 | **N** | 1,7 | 8,5 | 9,7 | 4,4 | 5,7 | 9,5 | 10, 4 | 8,7 | 9,7 | 10,4 | 2,2 | 11,6 | **S** |
| | | n | n | n | n | n | n | n | n | n | n | n | s | |
| mdb007 | **N** | 1,3 | 2,3 | 6,8 | 6,12 | 5,8 | 9,8 | 7,11 | 8,8 | 9,8 | 10,1 | 11,3 | 12,3 | **N** |
| | | n | n | n | n | n | n | n | n | n | n | n | n | |
| mdb010 | **S** | 1,12 | 2,12 | 6,2 | 7,6 | 5,5 | 6,4 | 7,6 | 8,5 | 9,5 | 10,6 | 12,10 | 12,12 | **N** |
| | | n | n | n | n | n | n | n | n | n | n | n | n | |
| mdb023 | **S** | 10,3 | 2,1 | 3,8 | 7,1 | 5,10 | 6,10 | 7,1 | 2,11 | 6,3 | 7,1 | 8,1 | 9,8 | **S** |
| | | n | n | n | s | n | n | s | n | n | s | s | n | |
| mdb028 | **S** | 1,7 | 2,12 | 6,7 | 7,7 | 5,9 | 9,5 | 10,4 | 5,7 | 12,7 | 10,5 | 11,5 | 9,5 | **S** |
| | | n | n | n | n | s | n | n | n | n | n | n | n | |
| mdb011 | **N** | 10,11 | 2,1 | 3,8 | 7,1 | 5,10 | 6,10 | 10,10 | 8,11 | 12,8 | 10,3 | 7,1 | 3,7 | **S** |
| | | n | n | n | s | n | n | n | n | n | n | s | n | |
| mdb014 | **N** | 1,7 | 5,6 | 3,7 | 7,4 | 8,12 | 6,7 | 7,4 | 8,5 | 9,5 | 10,7 | 11,2 | 9,12 | **S** |
| | | n | n | n | n | s | n | n | n | n | n | n | n | |
| mdb016 | **N** | 4,12 | 5,12 | 12,1 | 4,12 | 8,9 | 12,1 | 4,12 | 8,5 | 12,7 | 7,1 | 11,6 | 12,2 | **S** |
| | | s | s | n | s | n | n | s | n | n | s | s | s | |
| mdb015 | **S** | 10,11 | 5,10 | 6,11 | 7,1 | 5,11 | 3,1 | 7,1 | 8,11 | 6,1 | 4,11 | 6,4 | 3,12 | **S** |
| | | n | n | n | s | s | n | s | n | n | n | n | n | |
| mdb017 | **S** | 6,12 | 5,3 | 2,6 | 7,10 | 5,3 | 9,1 | 6,12 | 8,1 | 9,1 | 4,1 | 11,10 | 11,6 | **S** |
| | | n | n | n | n | n | s | n | s | s | n | n | s | |
| mdb019 | **S** | 1,10 | 2,1 | 6,3 | 11,11 | 5,11 | 3,10 | 2,10 | 8,7 | 6,10 | 2,3 | 8,7 | 12,8 | **S** |
| | | n | n | n | n | s | n | n | n | n | n | n | n | |
| mdb095 | **S** | 7,1 | 2,1 | 9,10 | 4,3 | 5,1 | 6,8 | 7,1 | 5,11 | 9,8 | 7,11 | 11,8 | 12,10 | **S** |
| | | s | n | s | n | s | n | s | s | n | n | n | n | |
| mdb102 | **S** | 1,12 | 2,12 | 12,1 | 7,6 | 5,6 | 12,4 | 1,12 | 2,12 | 12,4 | 1,8 | 9,7 | 12,4 | **N** |
| | | n | n | n | n | n | n | n | n | n | n | n | n | |
| mdb092 | **S** | 7,6 | 5,9 | 6,7 | 4,12 | 5,6 | 6,2 | 4,12 | 8,6 | 6,2 | 10,6 | 9,7 | 6,2 | **S** |
| | | n | s | n | s | n | n | s | s | n | n | n | n | |

Table 4: Results of using curvilinear modeling and PCA with Euclidean distance. Note: first number indicates the block number and second one signifies the image number in training set.

| Testing set | | Segments | | | | | | | | | | | | Image is |
|---|---|---|---|---|---|---|---|---|---|---|---|---|---|---|
| | | 1 | 2 | 3 | 4 | 5 | 6 | 7 | 8 | 9 | 10 | 11 | 12 | |
| mdb005 | **S** | 8,7 | 2,9 | 8,11 | 8,4 | 8,9 | 5,10 | 8,4 | 8,5 | 5,10 | 8,7 | 11,11 | 5,11 | **S** |
| | | n | n | n | n | n | n | n | n | n | n | n | s | |
| mdb006 | **N** | 1,2 | 2,2 | 3,6 | 7,2 | 8,9 | 6,6 | 4,12 | 8,7 | 6,6 | 10,9 | 11,7 | 12,6 | **S** |
| | | n | n | n | s | n | n | s | n | n | n | n | n | |
| mdb007 | **N** | 1,3 | 2,10 | 3,10 | 4,1 | 5,10 | 6,3 | 4,3 | 8,8 | 9,8 | 10,11 | 11,3 | 12,8 | **N** |
| | | n | n | n | n | n | n | n | n | n | n | n | n | |
| mdb010 | **S** | 1,6 | 2,12 | 3,12 | 4,12 | 5,12 | 9,4 | 4,12 | 8,5 | 9,4 | 10,6 | 11,6 | 12,2 | **S** |
| | | n | n | n | s | s | n | s | n | n | n | s | s | |
| mdb023 | **S** | 1,1 | 2,10 | 3,3 | 4,8 | 5,10 | 6,8 | 4,8 | 8,8 | 6,8 | 10,3 | 11,8 | 12,8 | **N** |
| | | n | n | n | n | n | n | n | n | n | n | n | n | |
| mdb028 | **S** | 1,4 | 2,12 | 3,4 | 4,4 | 5,2 | 9,7 | 7,2 | 8,7 | 9,7 | 10,2 | 11,7 | 12,7 | **S** |
| | | n | n | n | n | n | n | s | n | n | n | n | n | |
| mdb011 | **N** | 1,2 | 2,1 | 3,3 | 7,11 | 5,10 | 6,10 | 7,3 | 8,11 | 9,3 | 10,3 | 11,1 | 12,10 | **N** |
| | | n | n | n | n | n | n | n | n | n | n | n | n | |
| mdb014 | **N** | 1,9 | 2,7 | 3,7 | 4,7 | 5,5 | 9,4 | 7,9 | 8,5 | 9,5 | 10,9 | 11,2 | 12,6 | **S** |
| | | n | n | n | n | n | n | n | n | n | n | s | n | |
| mdb016 | **N** | 1,12 | 2,2 | 3,11 | 7,12 | 8,9 | 6,4 | 7,12 | 8,5 | 9,5 | 10,10 | 11,6 | 12,6 | **S** |
| | | n | n | n | s | n | n | s | n | n | n | s | n | |
| mdb015 | **S** | 1,1 | 2,11 | 3,11 | 4,1 | 8,8 | 9,11 | 4,3 | 8,11 | 9,1 | 10,1 | 11,1 | 12,4 | **S** |
| | | n | n | n | n | n | n | n | n | s | n | n | n | |
| mdb017 | **S** | 1,1 | 2,1 | 3,6 | 4,1 | 5,8 | 6,6 | 4,1 | 8,1 | 6,6 | 10,10 | 11,10 | 12,4 | **S** |
| | | n | n | n | n | n | n | n | s | n | n | n | n | |
| mdb019 | **S** | 1,10 | 2,1 | 3,3 | 7,3 | 5,1 | 6,8 | 4,6 | 8,7 | 6,3 | 10,5 | 11,7 | 12,8 | **S** |
| | | n | n | n | n | s | n | n | n | n | n | n | n | |
| mdb095 | **S** | 1,6 | 2,10 | 3,3 | 4,3 | 5,8 | 9,10 | 4,1 | 8,8 | 9,3 | 10,10 | 11,8 | 12,3 | **S** |
| | | n | n | n | n | n | s | n | n | n | n | n | n | |
| mdb102 | **S** | 1,6 | 2,12 | 3,5 | 4,12 | 5,6 | 6,4 | 4,6 | 8,6 | 9,4 | 10,6 | 11,6 | 12,4 | **S** |
| | | n | n | n | s | n | n | n | s | n | n | s | n | |
| mdb092 | **S** | 1,12 | 2,2 | 3,12 | 7,12 | 5,12 | 6,4 | 7,12 | 8,6 | 6,4 | 10,6 | 11,2 | 12,7 | **S** |
| | | n | n | n | s | s | n | s | s | n | n | s | n | |

Table 5: Results of using curvilinear modeling and PCA with Chebyshev distance. Note: first number indicates the block number and second one signifies the image number in training set.

| Testing set | | Segments | | | | | | | | | | | | Image is |
|---|---|---|---|---|---|---|---|---|---|---|---|---|---|---|
| | | 1 | 2 | 3 | 4 | 5 | 6 | 7 | 8 | 9 | 10 | 11 | 12 | |
| mdb005 | **S** | 8,7<br>n | 2,9<br>n | 8,11<br>n | 8,4<br>n | 8,9<br>n | 5,10<br>n | 8,4<br>n | 8,5<br>n | 5,10<br>n | 8,7<br>n | 11,11<br>n | 5,1<br>s | **S** |
| mdb006 | **N** | 1,2<br>n | 2,2<br>n | 3,9<br>n | 4,12<br>s | 8,9<br>n | 6,6<br>n | 4,12<br>s | 8,7<br>n | 6,6<br>n | 10,8<br>n | 11,7<br>n | 12,6<br>n | **S** |
| mdb007 | **N** | 1,3<br>n | 2,10<br>n | 3,10<br>n | 4,1<br>n | 5,10<br>n | 6,3<br>n | 7,1<br>s | 8,8<br>n | 9,8<br>n | 10,11<br>n | 11,3<br>n | 12,8<br>n | **S** |
| mdb010 | **S** | 1,6<br>n | 2,12<br>n | 3,12<br>n | 4,12<br>s | 5,12<br>s | 9,4<br>n | 7,6<br>n | 8,5<br>n | 9,4<br>n | 10,6<br>n | 11,6<br>s | 12,2<br>s | **S** |
| mdb023 | **S** | 1,1<br>n | 2,10<br>n | 3,3<br>n | 4,8<br>n | 5,10<br>n | 6,8<br>n | 4,8<br>n | 8,8<br>n | 6,8<br>n | 10,3<br>n | 11,8<br>n | 12,8<br>n | **N** |
| mdb028 | **S** | 1,4<br>n | 2,2<br>n | 3,4<br>n | 4,4<br>n | 5,2<br>n | 9,7<br>n | 7,2<br>s | 8,7<br>n | 9,7<br>n | 10,2<br>n | 11,7<br>n | 12,5<br>n | **S** |
| mdb011 | **N** | 1,2<br>n | 2,1<br>n | 3,3<br>n | 7,11<br>n | 5,10<br>n | 6,10<br>n | 7,3<br>n | 8,11<br>n | 6,9<br>n | 10,3<br>n | 11,1<br>n | 12,10<br>n | **N** |
| mdb014 | **N** | 1,9<br>n | 2,7<br>n | 3,7<br>n | 4,7<br>n | 5,12<br>s | 9,4<br>n | 4,7<br>n | 8,5<br>n | 9,5<br>n | 10,7<br>n | 11,2<br>s | 12,6<br>n | **S** |
| mdb016 | **N** | 1,11<br>n | 2,2<br>n | 3,11<br>n | 7,12<br>s | 8,9<br>n | 6,4<br>n | 7,12<br>s | 8,7<br>n | 9,5<br>n | 10,1<br>n | 11,6<br>s | 12,6<br>n | **S** |
| mdb015 | **S** | 1,1<br>n | 2,11<br>n | 3,11<br>n | 4,1<br>n | 8,8<br>n | 9,11<br>n | 4,3<br>n | 2,11<br>n | 9,1<br>s | 10,1<br>n | 11,6<br>s | 12,4<br>n | **S** |
| mdb017 | **S** | 1,1<br>n | 2,1<br>n | 3,6<br>n | 4,1<br>n | 5,8<br>n | 6,6<br>n | 4,1<br>n | 8,10<br>s | 6,6<br>n | 10,1<br>n | 11,10<br>n | 12,4<br>n | **S** |
| mdb019 | **S** | 1,3<br>n | 2,1<br>n | 3,3<br>n | 7,3<br>n | 5,1<br>s | 6,8<br>n | 4,12<br>s | 8,7<br>n | 6,3<br>n | 10,5<br>n | 11,7<br>n | 12,8<br>n | **S** |
| mdb095 | **S** | 1,6<br>n | 2,3<br>n | 3,3<br>n | 4,3<br>n | 5,8<br>n | 9,10<br>s | 4,1<br>n | 8,8<br>n | 9,3<br>n | 10,1<br>n | 11,8<br>n | 12,3<br>n | **S** |
| mdb102 | **S** | 1,6<br>n | 2,12<br>n | 3,5<br>n | 4,12<br>s | 5,6<br>n | 6,4<br>n | 4,6<br>n | 8,6<br>s | 6,7<br>n | 10,6<br>n | 11,2<br>s | 12,4<br>n | **S** |
| mdb092 | **S** | 1,12<br>n | 2,2<br>n | 3,12<br>n | 7,12<br>s | 5,12<br>s | 6,4<br>n | 7,12<br>s | 8,6<br>s | 6,4<br>n | 10,6<br>n | 11,2<br>s | 12,7<br>n | **S** |

In this work, two different approaches are employed for the detection of suspicious tissues in mammograms utilizing local PCA with data modeling. Linear modeling, local PCA, and Euclidean distance algorithm showed better results compared to Chebyshev distance. Euclidean measure produces in general less false detections. However, since the nature of cancerous tissues is more circular, Hough transform was employed to search for curved shapes rather than straight edges. The Hough modeling of the data combined with local PCA, and Euclidean distance produces improved results. In all, all algorithms produced over 90% correct classifications of blocks. However, the algorithm has several parameters that can be investigated for improvements. Thus future work is planned for an investigation of the performance dependency on the Hough parameters, threshold for the principal components, and $T$ which is a function of $\lambda$. The algorithm would improve if thresholding is optimized by using adaptive measures. Also, the distance measure is an important component of this framework, an optimum distance or even other similarity measures should be investigated. Moreover, block size is crucial for the performance and if smaller block size is used into the algorithm, the algorithm performance should improve, and hopefully, identify abnormalities in smaller size of neighborhoods down to pixel abnormality detection.

### ACKNOWLEDGMENTS

Partial support of this work was provided by the National Science Foundation grant MRI-0215356 and by Western Michigan University FRACASF award. The authors would like also to acknowledge Western Michigan University for its support and contributions to the Information Technology and Image Analysis (ITIA) Center.

### REFERENCES

[1] C. C. Boring, T. Squires, T. Tong, and S. Montgomery, "Cancer statistics," *Ca-A Cancer Journal for Clinicians*, vol. 44, no. 1, pp. 7–26, 1994.

[2] C. R. Smart, R. E. Hendrick, J. H. Rutledge III, and R. A. Smith, "Benefit of mammography screening in women ages 40 to 49 years: current evidence from randomized controlled trials," *Cancer*, vol. 75, no. 7, pp. 1619–1626, 1995.

[3] R. Zwiggelaar, "Separating background texture and image structure in mammograms," in *Proceedings of the British Machine Vision Conference (BMVC '99)*, pp. 362–371, British Machine Vision Association, Nottingham, UK, September 1999.

[4] H.-P. Chan, B. Sahiner, N. Patrick, et al., "Computerized classification of malignant and benign microcalcifications on mammograms: texture analysis using an artificial neural network," *Physics in Medicine and Biology*, vol. 42, no. 3, pp. 549–567, 1997.

[5] G. Lemaur, K. Drouiche, and J. DeConinck, "Highly regular wavelets for the detection of clustered microcalcifications in mammograms," *IEEE Transactions on Medical Imaging*, vol. 22, no. 3, pp. 393–401, 2003.

[6] W. M. Morrow, R. B. Paranjape, R. M. Rangayyan, and J. E. L. Desautels, "Region-based contrast enhancement of mammograms," *IEEE Transactions on Medical Imaging*, vol. 11, no. 3, pp. 392–406, 1992.

[7] L. Shen, R. M. Rangayyan, and J. E. L. Desautels, "Application of shape analysis to mammographic calcifications," *IEEE Transactions on Medical Imaging*, vol. 13, no. 2, pp. 263–274, 1994.

[8] T. C. Wang and N. B. Karayiannis, "Detection of microcalcifications in digital mammograms using wavelets," *IEEE Transactions on Medical Imaging*, vol. 17, no. 4, pp. 498–509, 1998.

[9] C. Y. Chen, C. C. Chang, and R. C. T. Lee, "A near pattern-matching scheme based upon principal component analysis," *Pattern Recognition Letters*, vol. 16, no. 4, pp. 339–345, 1995.

[10] B. Pinkowski, "Principal component analysis of speech spectrogram images," *Pattern Recognition*, vol. 30, no. 5, pp. 777–787, 1997.

[11] Z. Ye and G. Auner, "Principal component analysis approach for biomedical sample identification," in *Proceedings of IEEE International Conference on Systems, Man, and Cybernetics (SMC '04)*, pp. 1348–1353, The Hague, The Netherlands, October 2004.

[12] R. Swiniarski and A. Swiniarsk, "Comparison of feature extraction and selection methods in mammogram recognition," *Annals of the New York Academy of Sciences*, vol. 980, no. 12, pp. 116–124, 2002.

[13] I. Abdel-Qader, A. Kawshif, O. Abudayyeh, and D. Miller, "Feature extraction using pca and cluster analysis," in *Proceedings of the 4th IEEE Electro/Information Technology Conference*, Indianapolis, Ind, USA, June 2003.

[14] M. Brejl and M. Sonka, "Directional 3D edge detection in anisotropic data: detector design and performance assessment," *Computer Vision and Image Understanding*, vol. 77, no. 2, pp. 84–110, 2000.

[15] S. Duan and Q.-Q. Qin, "Edge detection based on dynamic morphology," in *MIPPR 2005: Image Analysis Techniques*, D. Li and H. Ma, Eds., vol. 6044 of *Proceedings of SPIE*, Wuhan, China, October-November 2005.

[16] A. Goshtasby and H.-L. Shyu, "Edge detection by curve fitting," *Image and Vision Computing*, vol. 13, no. 3, pp. 169–177, 1995.

[17] O. A. Zuniga and R. M. Haralick, "Integrated directional derivative gradient operator," *IEEE Transactions on Systems, Man and Cybernetics*, vol. 17, no. 3, pp. 508–517, 1987.

[18] W. E. Higgins and C. Hsu, "Edge detection using two-dimensional local structure information," *Pattern Recognition*, vol. 27, no. 2, pp. 277–294, 1994.

[19] S. Zhan and R. Mehrotra, "Zero-crossing-based optimal three-dimensional edge detector," *Computer Vision, Graphics, and Image Processing: Image Understanding*, vol. 59, no. 2, pp. 242–253, 1994.

[20] R. Mehrotra and S. Zhan, "A computational approach to zero-crossing-based two-dimensional edge detection," *Graphical Models and Image Processing*, vol. 58, no. 1, pp. 1–17, 1996.

[21] M. Song, X.-L. Hu, and Y. Gao, "Image edge detection based on omnidirectional multiscale morphology," *Journal of Yangzhou University(Natural Science Edition)*, vol. 8, no. 1, pp. 39–42, 2005.

[22] P. Hough, "Methods and means for recognizing complex patterns," US patent no. 069654, 1962.

[23] R. Duda and P. Hart, "Use of the hough transformation to detect lines and curves in pictures," *Communications of the ACM*, vol. 15, no. 1, pp. 11–15, 1972.

[24] J. Illingworth and J. Kittler, "A survey of the hough transform," *Computer Vision, Graphics, & Image Processing*, vol. 44, no. 1, pp. 87–116, 1988.

[25] V. F. Leavers, "Which hough transform?" *Computer Vision, Graphics, and Image Processing: Image Understanding*, vol. 58, no. 2, pp. 250–264, 1993.

[26] T. Rabbani and F. van den Heuvel, "Efficient hough transform for automatic detection of cylinders in point clouds," in *Proceedings of the 11th Annual Conference of the Advanced School for Computing and Imaging (ASCI '05)*, Het Heijderbos, Heijen, The Netherlands, June 2005.

[27] C. F. Olson, "Constrained hough transforms for curve detection," *Computer Vision and Image Understanding*, vol. 73, no. 3, pp. 329–345, 1999.

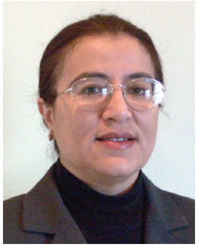

**Ikhlas Abdel-Qader** is currently an Associate Professor of Electrical and Computer Engineering, and is the Founder and Director of the Information Technology and Image Analysis (ITIA) Center in the College of Engineering and Applied Sciences at Western Michigan University. She earned her Ph.D. degree in electrical and computer engineering from North Carolina State University, Raleigh, in 1992. Her research expertise and interests include feature extraction, motion estimation, and image compression with applications in medical imaging, nondestructive testing, and intelligent transportation systems. She is also actively involved in promoting engineering careers and research to undergraduate students as well as to middle and high school students. She is a Member of the Institute of Electrical and Electronic Engineers (IEEE); the IEEE Acoustics, Speech, and Signal Processing Society; and the IEEE Engineering in Medicine and Biology Society. She is also a Member of the Society of Women Engineers, a Member of the Honor Society of Phi Kappa Phi, and an Associate Member of the Sigma Xi Scientific Research Society. She is a registered Professional Engineer in the State of Minnesota since 1996.

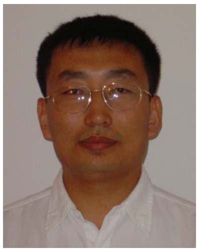

**Lixin Shen** received his B.S. and M.S. degrees from Peking University, China, in 1987 and 1990, respectively, and his Ph.D. degree from Zhongshan University, China, in 1996, all in mathematics. From September 1996 to July 2001, he was a Research Fellow at Center for Wavelets, Approximation, and Information Processing, National University of Singapore. From August 2001 to July 2002, he was a Post-Doctoral Fellow at Virtual Environments Research Institute, University of Houston. From August 2002 to July 2004, he was with the Department of Mathematics, West Virginia University. He is currently an Assistant Professor at the Department of Mathematics, Western Michigan University. His current research interests are wavelets and their application in image processing.

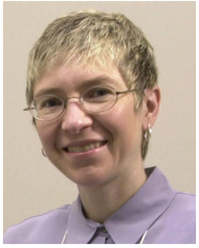

**Christina Jacobs** received her B.S. degree in neuroscience from University of Rochester in Rochestor, New York, in 1986 and her M.D. degree in medicine from New York University in New York City, in 1991. She is board certified in radiology from the American Board of Radiology since 1996. She is currently a Member of Advanced Radiology Services and serves as the Medical Director of Bronson Center for Women in Kalamazoo, Michigan. Her research interests include mammography and ovarian cancer detection using CT imaging. She is a Member of the Society of Breast Imaging, the Radiological Society of North America, the American College of Radiology, the American Association for Women Radiologists, and Michigan State Medical Society.

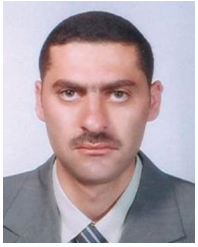

**Fadi Abu Amara** received his Computer Engineering bachelor degree from Faculty of Engineering Technology, Al-Balqa' Applied University, in Jordan, in 2001. Currently he is a Graduate Student in the Department of Electrical and Computer Engineering and a Research Assistant in the Information Technology and Image Analysis Center at Western Michigan University. His area of interests includes medical image analysis, fuzzy logic, and neural network.

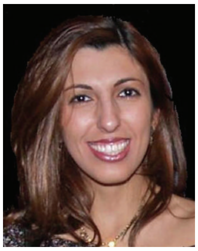

**Sarah Pashaie-Rad** received the B.S. degree from Sharif University of Technology and the M.S. degree, with highest honor, from Western Michigan University, both in electrical engineering. Her graduate research interests involved digital image processing focused on medical applications. She joined St. Jude Medical, Inc. in September 2005. Her current responsibilities include test failure analysis for implantable cardioverter defibrillators (ICD).

